# Forearm Compartment Syndrome Following a Thermal-Crush Injury Treated With a Dorsal Ulnar Artery Flap: A Case Report

**DOI:** 10.7759/cureus.109119

**Published:** 2026-05-18

**Authors:** Orson R Juan-Hernández, Guillermo B Gómez-Morales, Carlos A Luna-Morales, Frida I Rosas-Lezama

**Affiliations:** 1 General Surgery, Hospital General de Zona No. 2, Instituto Mexicano del Seguro Social (IMSS), Tuxtla Gutiérrez, MEX; 2 Internal Medicine, Hospital General Regional No. 12 “Lic. Benito Juárez García”, Instituto Mexicano del Seguro Social (IMSS), Mérida, MEX

**Keywords:** burns, compartment syndromes, crush injuries, debridement, fasciotomy, forearm injuries, ischemia, laundering, surgical flaps

## Abstract

Compartment syndrome (CS) is a condition that requires immediate attention; if diagnosis is delayed and treatment is not provided promptly, it can lead to complications resulting in permanent functional disability or limb loss. As the name suggests, once a muscle compartment is affected by an injury, this syndrome may develop, manifesting as progressive inflammation and edema, leading to reduced blood flow to the limb. Diagnosis is primarily clinical, based on signs of impaired perfusion. Fasciotomy is the treatment of choice, helping to prevent amputations when performed in a timely manner. However, it is associated with significant complications, as closing the fasciotomy incision can pose a considerable surgical challenge; therefore, post-fasciotomy procedures are just as important as the decompression procedure itself. In this context, the use of grafts and delayed wound closure are frequent therapeutic options. When these measures fail, the use of flaps is feasible, although there is limited literature on their use following fasciotomy.

The purpose of this article is to present the case of a patient whose forearm became trapped in a dryer, resulting in an injury from a combined crush and thermal mechanism. Despite multiple procedures, coverage of the open wound was not achieved, so the use of flaps was considered. The radial forearm flap sacrifices one of the main arteries of the hand, the interosseous flap can be difficult to perform, and the inguinal flap requires prolonged immobilization and two procedures. Therefore, we opted for the dorsal ulnar flap to close the post-fasciotomy wound, which is quick and easy to perform, relies on a continuous blood vessel without compromising the major arteries of the hand, allows for early mobilization, and enabled us to achieve adequate coverage of the wound with tendon exposure involving the wrist and hand. We conclude that the dorsal ulnar flap is a reliable and effective option for wound coverage following fasciotomy in forearm CS.

## Introduction

Compartment syndrome (CS) is a medical emergency that has been recognized since ancient times. Wars, natural disasters, and industrial accidents provide the perfect environment for a person to suffer crush or thermal injuries to their limbs; these severe injuries lead to inflammation. As this inflammation progressively increases, blood flow gradually decreases, damaging all the structures within this closed space known as the compartment, which contains muscles that distribute the mechanical load, nerves, and vessels, all delimited by an inelastic fascia. This can go so far as to jeopardize the viability of the entire limb [1,2].

The intracompartmental pressure of the interstitial fluid is around 10 mmHg. When the intracompartmental pressure exceeds the 20-33 mmHg typically found in blood capillaries, a critical closure pressure occurs, causing the microcirculation to collapse and compromising distal capillary blood flow. This leads to a critical state of tissue hypoxia, with accumulation of metabolic waste products [1,3,4].

Muscle necrosis occurs after just three hours of continuous pressure, and after six hours, the prognosis may be compromised, since deficits in nerve conduction may occur; irreversible infarction of all skeletal muscle occurs after eight hours. Finally, pressure maintained for 12 hours may completely abolish nerve conduction or muscle response [1,5,6].

Complications of this process include myonecrosis and rhabdomyolysis, which manifest as infection, paralysis, and insensitivity due to permanent nerve damage, Volkmann's contracture, failure of fracture consolidation, and, in the most severe cases, loss of the limb requiring amputation [1,3,4,7].

The treatment of choice is fasciotomy, which involves making an incision in the fascia of the compartment that is under tension, thereby breaking the pathophysiological cycle caused by the abnormal increase in pressure. By cutting through the skin and fascia, along with eschar in the case of burns, these incisions allow the pressure to be released, resulting in an increase in muscle volume and the restoration of blood flow. Therefore, if this procedure is delayed, the limb is expected to be compromised as a result [1,7].

Although fasciotomy is the standard surgical treatment [1,2,6], closing the post-fasciotomy wound can be quite complex, since fasciotomy involves an incision from the skin down to the fascia, causing the muscles to protrude through the wound. While increased volume acts as a factor that widens the wound, the wound edges gradually heal, making fasciotomy closure a challenging procedure.

For forearm wounds following fasciotomy, delayed closure, the use of negative pressure therapy as a bridging measure, the use of grafts, and, less frequently, the use of flaps to achieve skin coverage have been described [1,6,8,9].

Among the flaps used to close wounds in the forearm and wrist, we would like to focus specifically on the dorsal ulnar flap, which is a regional forearm flap with independent circulation that does not require sacrifice of the main vascular trunks and does not require microsurgical techniques for its execution [6,10-13]. The dorsal ulnar flap is proposed as a procedure that can yield satisfactory results when other procedures have failed.

## Case presentation

**Figure 1:**
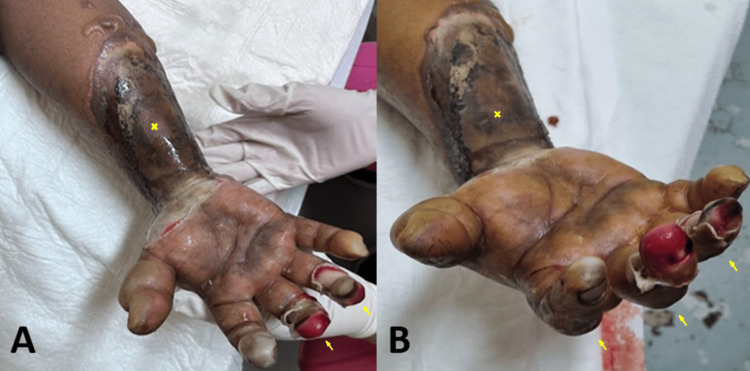
Clinical appearance 12 hours after the injury. A) The palmar region of the right forearm shows a third-degree burn (yellow X), with the burn extending to the wrist and hand. B) The phalanges appear pale (yellow arrows), with decreased capillary refill secondary to increased pressure.

A 41-year-old man suffered an accident in which his right forearm and arm were accidentally caught in an industrial dryer, resulting in a thermal-crush injury for 30 minutes, with a burn covering four percent of the body surface area on the limb. He went to the emergency department and was eventually diagnosed with a third-degree burn because the burn appeared gray, pale, and dry. He was re-evaluated 12 hours later because the pain worsened, and paresthesia, a feeling of tension, and a weak pulse began to appear, leading to the conclusion that the patient had CS (Figure 1).

**Figure 2:**
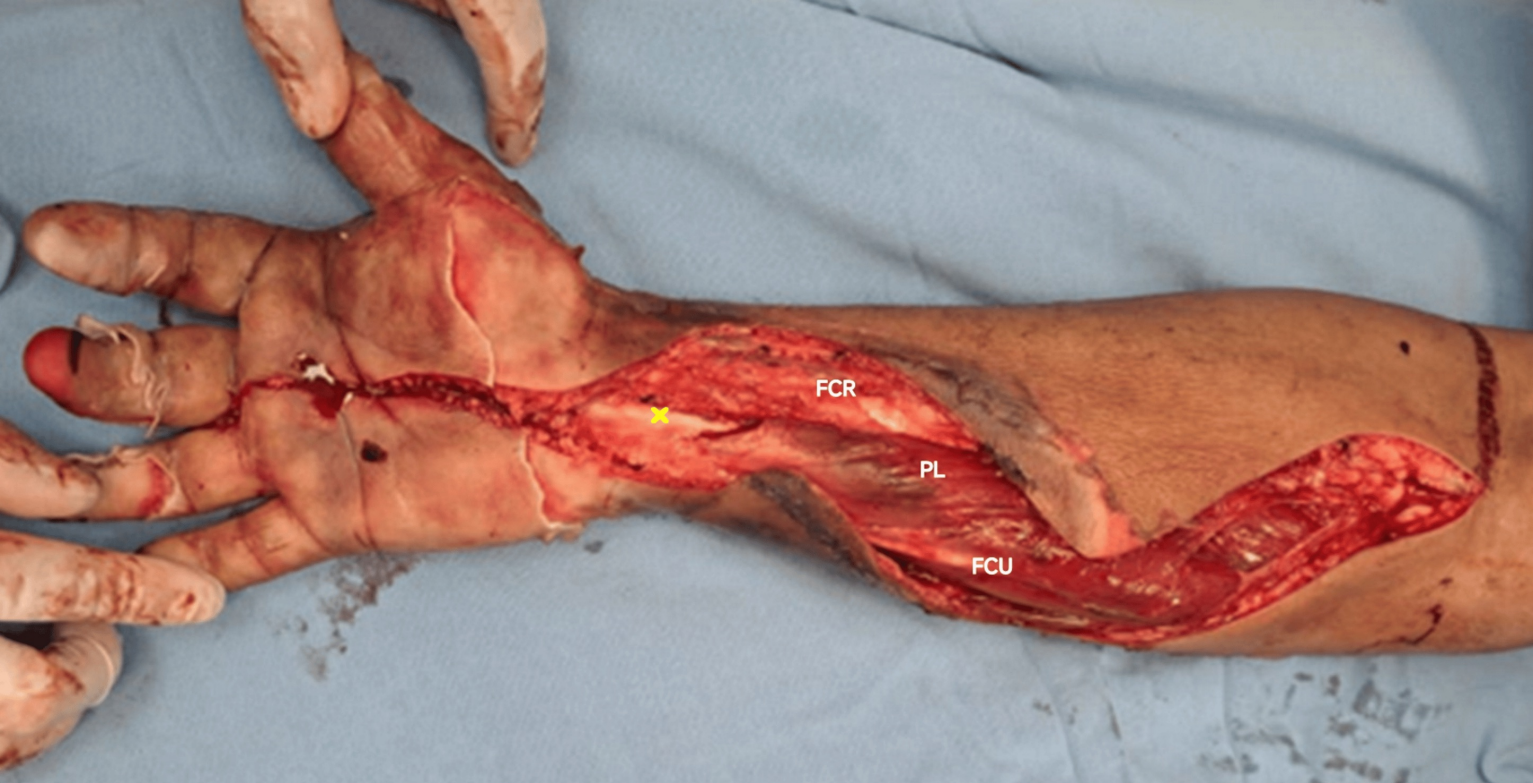
Postoperative appearance after fasciotomy. Fasciotomy of the right forearm releasing the anterior compartment; the flexor carpi radialis (FCR), palmaris longus (PL), and flexor carpi ulnaris (FCU) muscles are visible. There is slight tissue discoloration due to ischemia and pallor of the phalanges, with blisters. At the wrist, the flexor tendons (yellow X) are still partially preserved, with a slight pearly-white appearance, despite generalized lysis of the surrounding tissues.

It was decided to proceed to the operating room immediately to perform a tangential fascial release through an S-shaped incision (Figure 2). Intraoperative findings revealed tension in the compartments of the right forearm, with distal limb ischemia, decreased temperature, numbness, and delayed capillary refill.

**Figure 3:**
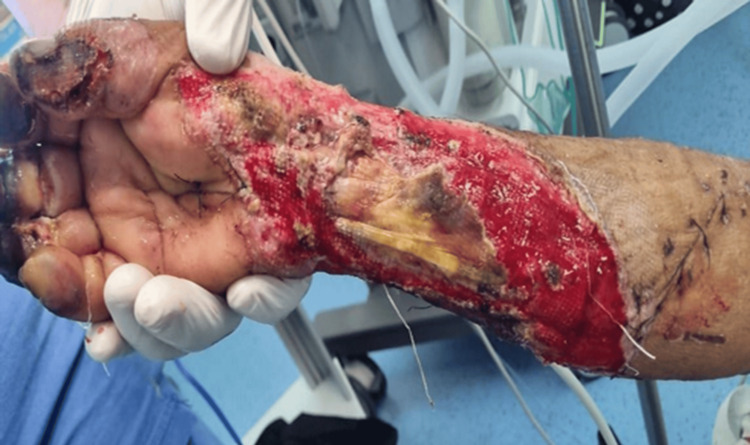
Appearance eight days after the injury before surgical debridement. Tendon exposure is observed. The tendon is desiccated, and peripheral granulation tissue with necrotic patches is present. Limitation in wrist range of motion is beginning to appear.

**Figure 4:**
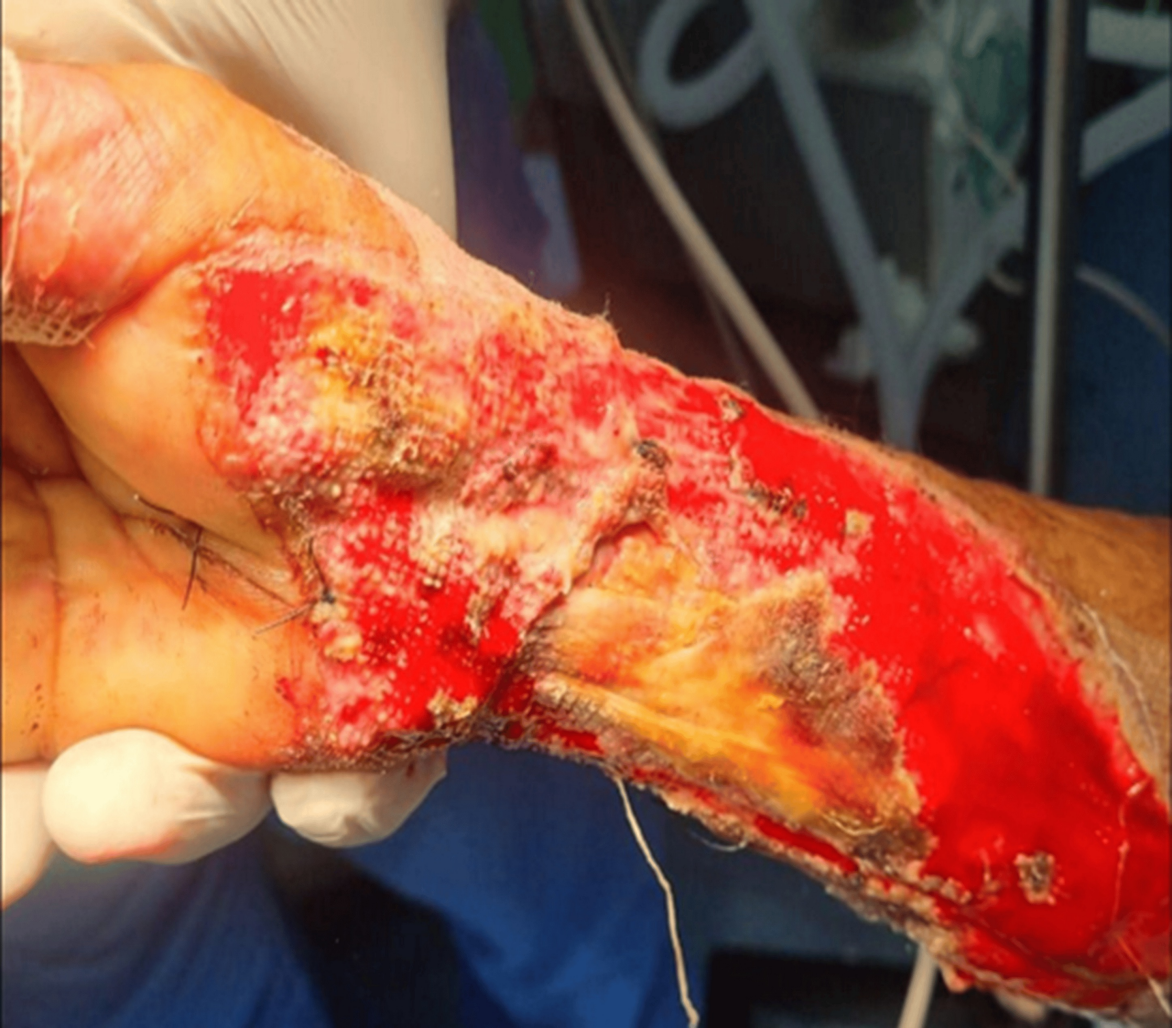
Appearance following surgical irrigation and debridement of necrotic areas. The surgical site is prepared for the placement of skin grafts in the next procedure. The necrotic patches are carefully debrided while preserving as much of the tendon structures as possible.

Subsequently, eight days later, distal blood flow and the presence of radial and ulnar pulses were identified; therefore, removal of necrotic tissue and debridement were performed, preparing the patient for skin graft placement. The wound culture was negative (Figures 3-4).

**Figure 5:**
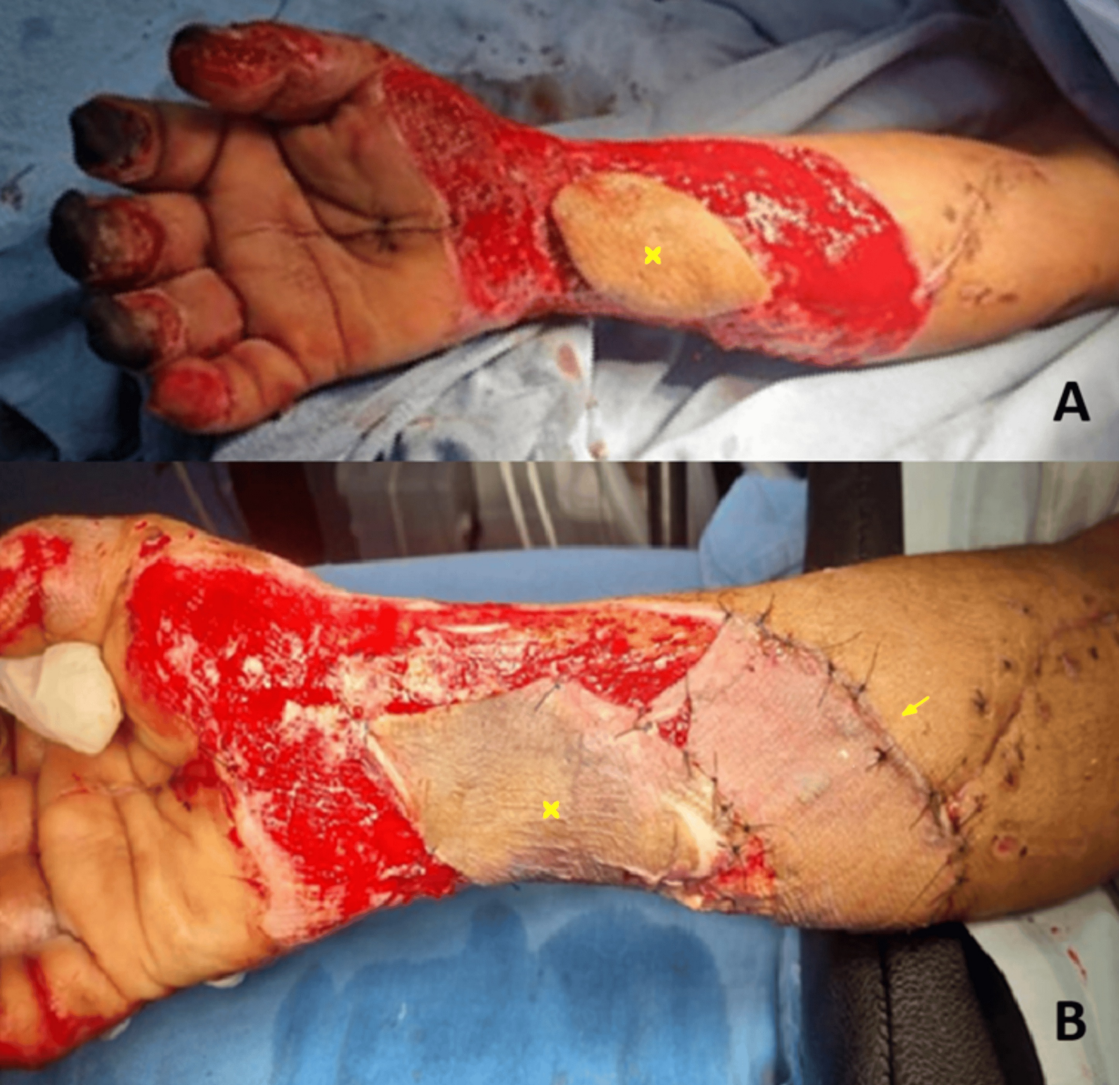
Appearance 16 days after the injury. A) Surgical wound with a full-thickness graft (yellow X) placed over the tendon surface, surrounded by abundant granulation tissue. B) Partial-thickness grafts (yellow arrow) are placed around the full-thickness graft (yellow X) in the proximal half of the wound.

**Figure 6:**
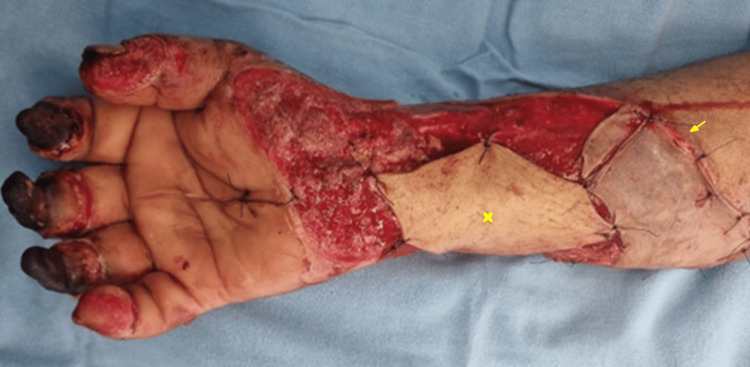
Appearance after placement of skin grafts and tendon coverage. Wound with bleeding granulation tissue, with partial-thickness grafts (yellow arrow) placed in the area distal to the wound, showing adequate coverage, and a full-thickness graft (yellow X) in the area of tendon exposure.

A new debridement was performed, and it was decided to harvest partial-thickness grafts using a Padgett dermatome (0.18-0.24 mm) from the right thigh to cover the exposed area, and a full-thickness graft (Wolfe-Krause graft) for the wrist area with tendon exposure; partial wound coverage was achieved (Figures 5-6).

**Figure 7:**
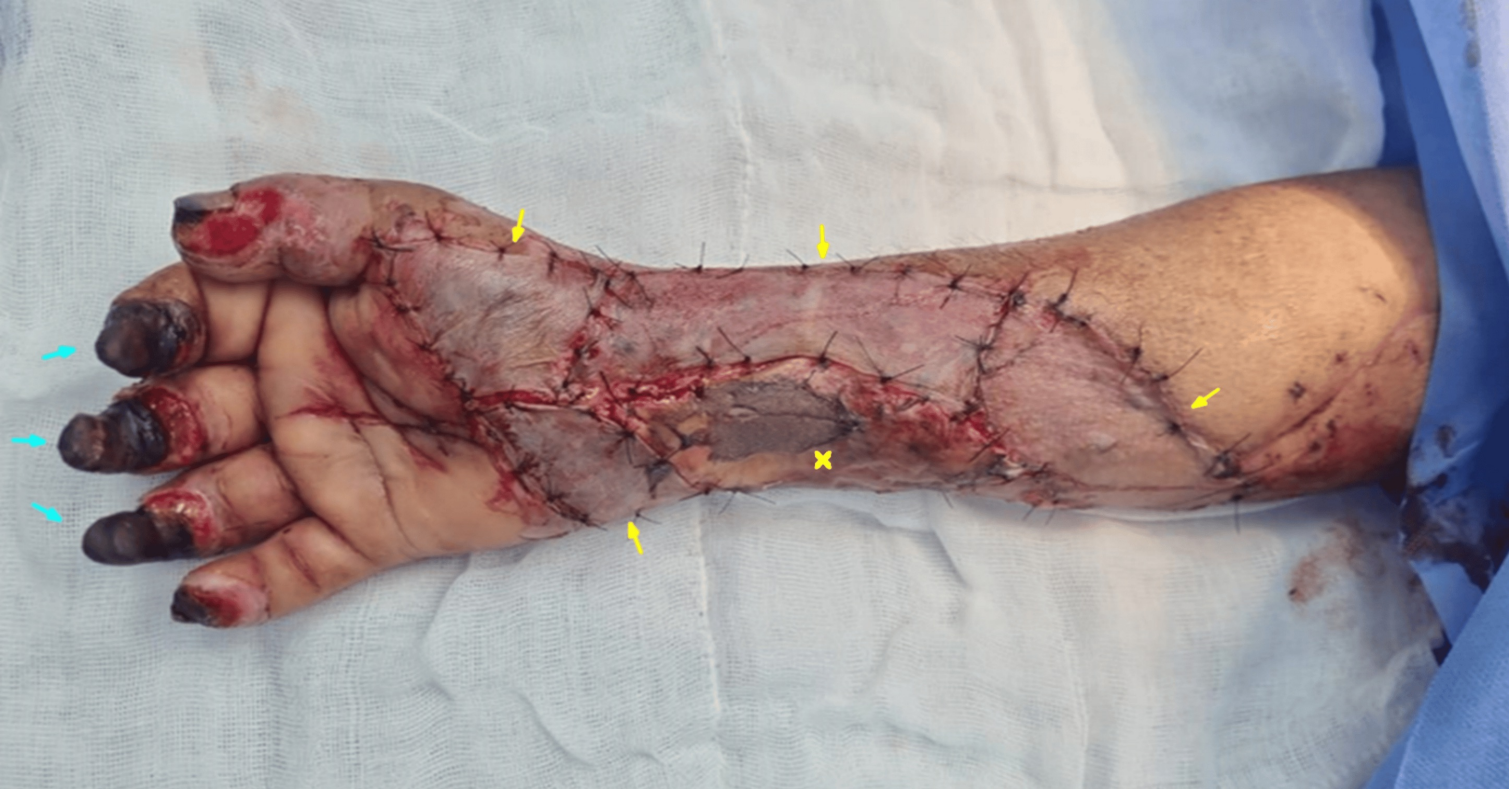
Appearance 20 days after the injury. Partial-thickness grafts have been placed over the entire wound (yellow arrows), and the overall appearance is good. The full-thickness graft (yellow X) still shows a color similar to that of the surrounding tissue. Necrosis of the distal phalanges is delineated (blue arrows).

Adequate integration of all grafts was observed on the volar surface of the forearm, as well as in the wrist and part of the hand, which were also covered with grafts, achieving coverage of the entire wound. However, necrosis of the distal phalanges began to appear, though no intervention was required at that time (Figure 7).

**Figure 8:**
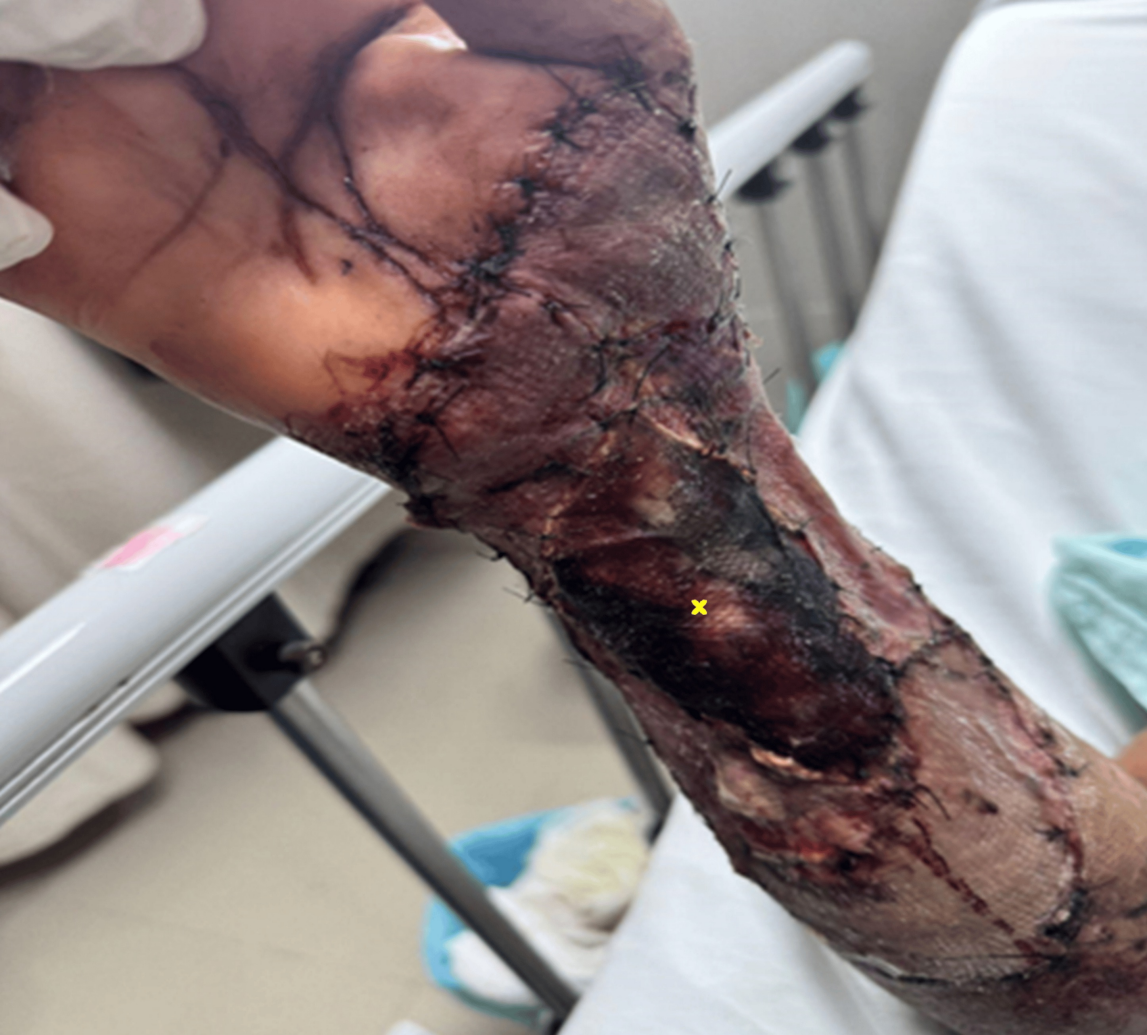
Appearance 28 days later, showing graft failure. There is evidence of necrosis of the full-thickness Wolfe-Krause graft (yellow X). Therefore, it was decided to remove it, and the wrist tendon was exposed again. The failure of this procedure necessitated considering a more appropriate surgical approach.

Subsequently, one week later, the wrist and hand were observed to have exudate, necrosis, and failure of the full-thickness graft to integrate, with subsequent tendon exposure (Figure 8); therefore, graft failure was concluded, and it was decided to remove it.

**Figure 9:**
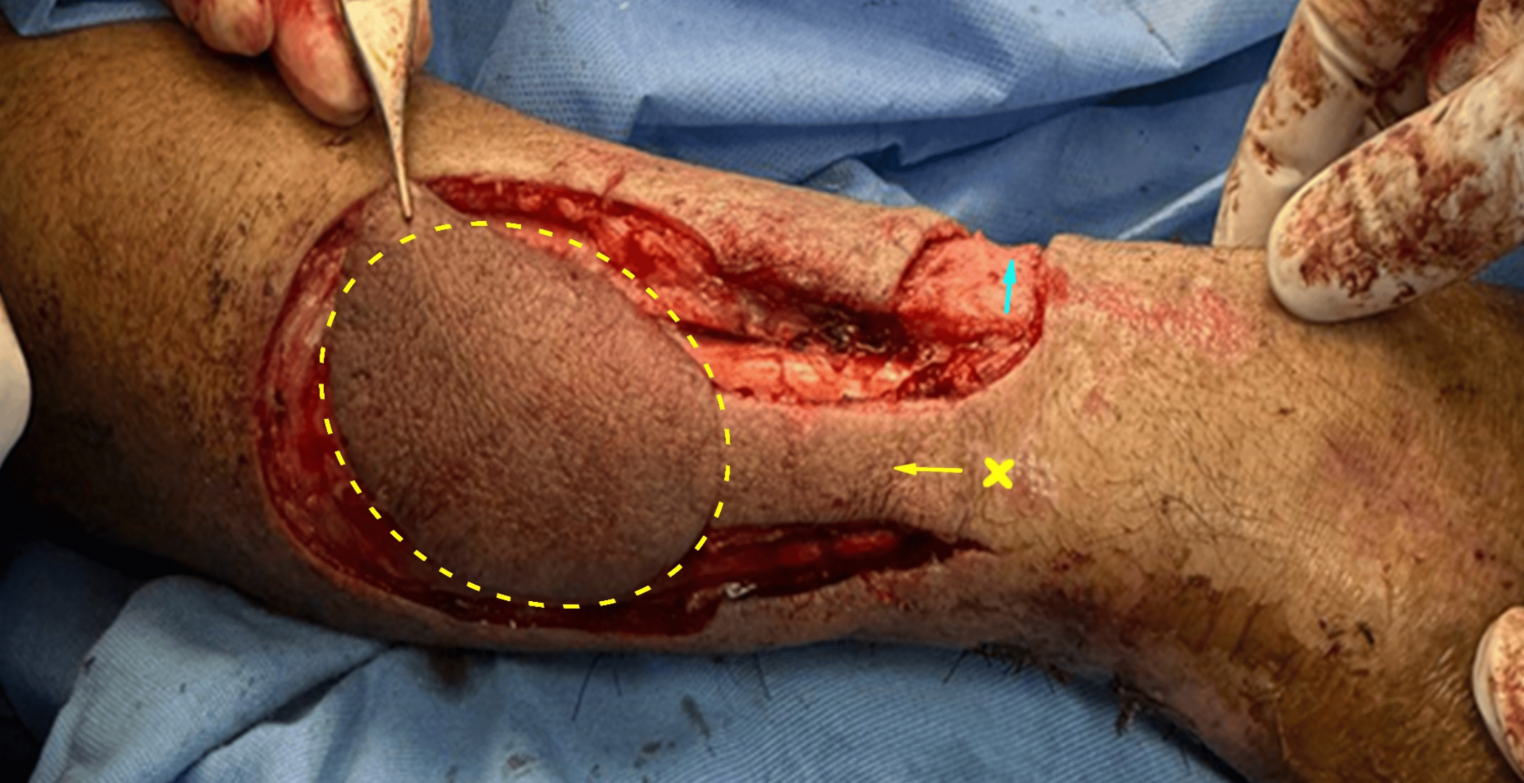
Surgical procedure to perform the dorsal ulnar flap on day 35. The dorsal ulnar flap is constructed in a peninsula shape. The flap components are exposed: a skin paddle (dotted yellow arrow), the flap pedicle (yellow arrow), and the recipient site (blue arrow). The flap will be rotated medially at its pivot point (yellow X) to cover the volar surface of the forearm.

**Figure 10:**
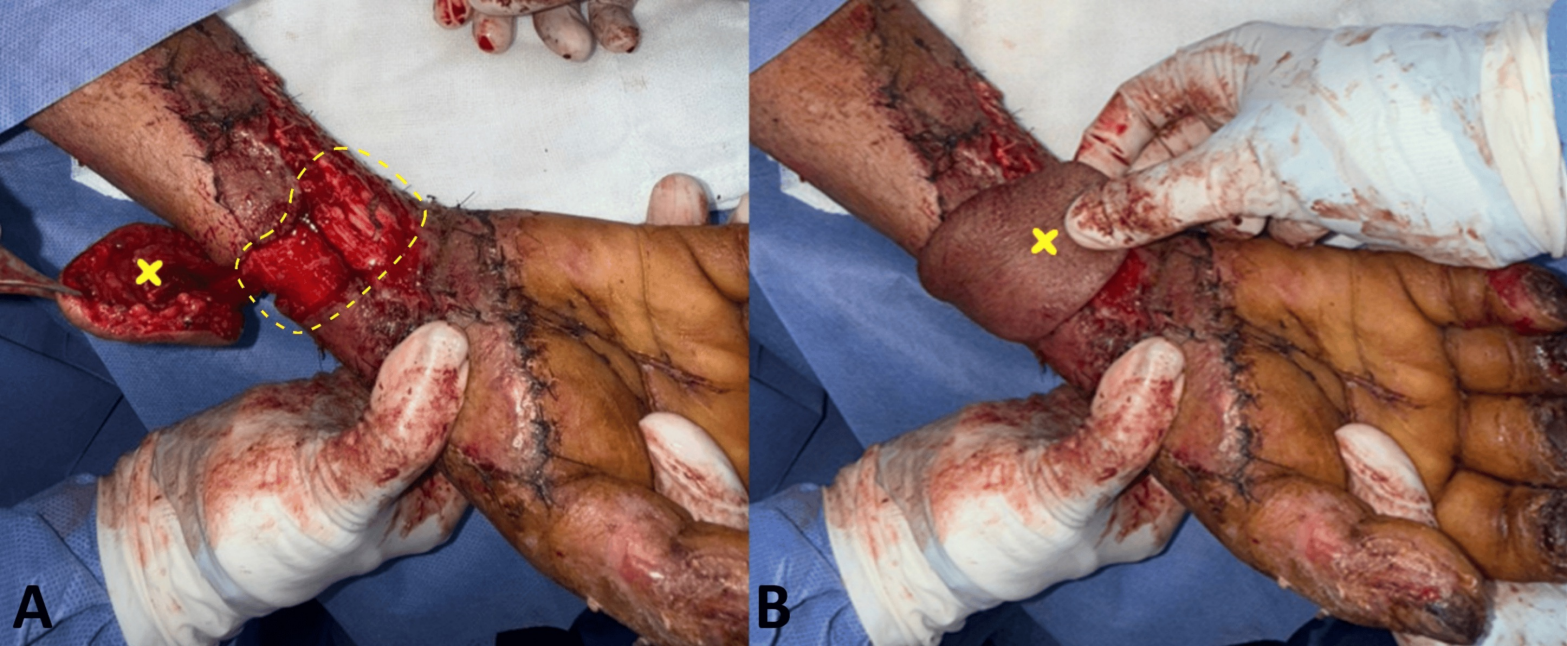
Mobilization of the dorsal ulnar flap during the surgical procedure. A) The recipient site on the wrist is visible, with the tendons exposed (yellow dotted arrow). These tendons are desiccated and free of necrotic tissue following debridement, and the dorsal ulnar flap is moved along with its respective skin paddle (yellow X). B) The flap (yellow X) is rotated at a right angle to cover the exposed tendons. The necrotic distal phalanges are also amputated.

Finally, after more than a month in the hospital, it was decided to perform a dorsal ulnar fasciocutaneous flap to cover the wound on the wrist and hand (Figures 9-10). The donor site of the dorsal flap was quite large, so primary closure was not possible. Therefore, the defect was covered with partial-thickness skin grafts harvested from the groin. In addition, the necrotic phalanges were amputated.

The patient was discharged after confirming tissue viability and with no evidence of a systemic inflammatory response.

**Figure 11:**
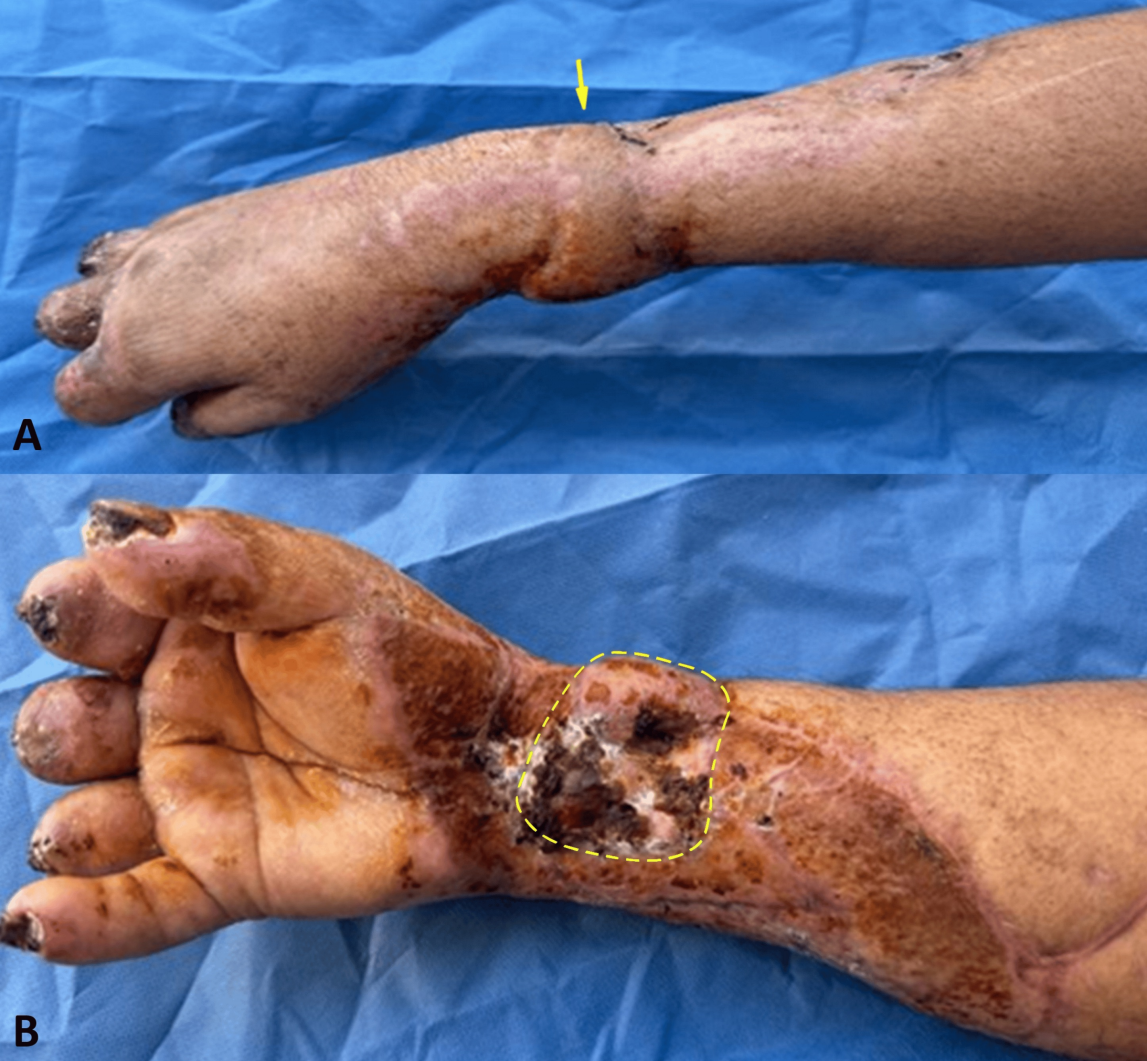
Two-month follow-up after hospital discharge. A) Dorsal view showing the wrist flexed at 30 degrees. The pivot point and pedicle of the flap are marked (yellow arrow). The flap wraps medially around the forearm. B) Palmar view showing the completely healed wound with the flap, delimited by the yellow dotted line, and the integrated skin grafts around it. Although full coverage was achieved, only thumb-to-little finger opposition was maintained. The radial and ulnar pulses are palpable.

Two months later, the patient was evaluated during a follow-up visit (Figure 11), where it was confirmed that the flap remained viable and had healed. Wrist flexion was partially preserved up to 30 degrees. Pinch function between the thumb and little finger was also maintained, and sensation in the hand was preserved. However, the sequelae were significant: first, due to the amputation of the necrotic phalanges, and second, due to sensory alterations resulting from the absence of sensation on the surface of the flap and decreased sensation on the palmar surface of the forearm. Therefore, it was decided to continue follow-up visits every three months while the patient continued therapy in the rehabilitation department.

## Discussion

Several factors are strongly associated with forearm CS. A study conducted by Kalyani BS et al. compiled 84 cases of acute CS; the primary causes identified were fractures (31%) and arterial injuries (10.7%), followed by narcotic use (9.5%), crush injuries (8.3%), infiltrations (8.3%), gunshot wounds (8.3%), and stab wounds (7.1%) [14]. Similarly, in a cohort of 119 patients who underwent forearm fasciotomies, Zhang D et al. found that 34% were related to fractures and the remaining 66% to soft tissue injuries [8]. In contrast to these findings, a systematic review conducted by Khoshhal KI et al. analyzed 83 articles involving a total of 684 individuals; the vast majority of these cases involved fractures (65.4%) and soft tissue injuries (30.7%). This latter group was further subdivided, with relative proportions attributed to burns (18.6%) and crush injuries (17.1%); only 4.7% of cases specifically involved the mechanism of the forearm being trapped in machinery [3].

Regarding the diagnosis of CS, it is primarily clinical; therefore, the clinician’s suspicion and physical examination are essential. The classic symptoms include disproportionate pain, which may be perceived as a burning sensation or deep pain within the arm; a sensation of pressure, which can be confirmed as a tense compartment upon palpation; paresthesia; paralysis; pallor; poikilothermia; and diminished pulses in the affected limb compared to the healthy limb [1,2].

The diagnosis of CS can be complex in certain scenarios when the clinical picture is inconclusive; therefore, compartment pressure measurements should be performed as soon as possible to diagnose and intervene promptly [1,2,8]. To measure pressure, clinicians can use the Stryker pressure monitor/needle manometer, the Whitesides needle method, the wick catheter, the slit catheter, and the intracompartmental catheter with a tip transducer. Near-infrared spectroscopy (NIRS) and laser Doppler flowmetry have been proposed as non-invasive measures; however, their effectiveness is not well established [1,2,6].

There are two key measurements obtained using continuous invasive methods: absolute compartment pressure and delta pressure. The first measurement is obtained directly using monitoring devices; the second is calculated by determining the diastolic pressure and subtracting the absolute compartment pressure. These pressures should be measured for at least a couple of hours. An absolute pressure >30 mmHg or a pressure differential <30 mmHg is considered diagnostic of CS [1,2,7].

Once diagnosed, ideally, early fasciotomy should be performed within the first eight hours, with a wide incision releasing the affected compartments [1,2]. In the case of a fasciotomy on the volar surface of the forearm, the incision must run along the entire surface of the forearm, with several curves, and its length should extend from the base of the arm to just beyond the wrist, including the carpal tunnel [1]. Following fasciotomy, the next most important step is debridement; these procedures must always be performed in the operating room, and several debridements, on average two, may be required before suturing the wound can even be considered. These debridements should involve the removal of devitalized tissue, specifically avascular, discolored, or non-contractile muscle [8,9]. The consensus regarding fasciotomies performed eight to 24 hours after trauma is less clear; however, they may be performed provided that clinicians remain vigilant for systemic and local complications and the need for reoperation. After 36 hours, fasciotomy is not recommended due to damage caused by substances released during ischemia-reperfusion. Even in very advanced cases (>72 hours), primary amputation may be considered [1,9].

Last but not least, regarding wound closure, skin coverage, and subsequent procedures, a systematic review of forearm CS conducted by Kalyani BS et al., which examined 89 cases, found that 73% of patients were treated with fasciotomy; of these, 61% required skin grafts and 39% required secondary closure [14]. In a study of 129 limbs, 40.4% required the use of flaps or grafts; however, the study does not specify which flaps were used or the exact number [9]. Some authors have reported even higher percentages; for example, Zhang D et al. reported in their series that 47% of forearms required skin grafts [8].

Although it has been reported that a post-fasciotomy wound may require grafts, the situation changes entirely when there is tendon exposure, and this must be taken into account when deciding on optimal management. With regard to the use of grafts, their application is not recommended when there is bone or tendon exposure unless the periosteum or paratenon is intact. A graft cannot survive outside these conditions [6]. We strongly recommend that, in the case of a wound with tendon exposure, the area be carefully examined for dull yellow or gray discoloration, a dry appearance, and the absence of granulation tissue on the tendon surface, since all these findings indicate an affected paratenon, and it is highly likely that a graft will fail, as occurred in our case.

For wounds involving tendon exposure with paratenon involvement, the use of flaps is preferred.

With regard to the use of flaps, numerous pedicled flaps have been described for the reconstruction of the forearm, wrist, and hand, thereby achieving skin coverage without the need for microsurgical procedures [11,13,15-17].

In our case, we opted for a dorsal ulnar flap, also known as a Becker-Gilbert flap [11], which is a fasciocutaneous flap based on the dorsal ulnar artery that preserves the main arterial axes of the hand, unlike the reverse radial flap; provides a substantial skin paddle; is easy to dissect compared to the reverse posterior interosseous flap [10,12,13,18]; allows for early mobilization of the hand because it is performed in a single procedure, unlike the inguinal flap or the thoracoepigastric flap [16,17]; allows early rehabilitation; and reduces limitations caused by scarring. Tables 1-2 summarize the advantages, disadvantages, and features of each of the pedicled flaps commonly used in reconstructive surgery of the forearm and hand.

**Table 1:** Pedicled flaps for coverage of defects in the distal forearm and wrist: advantages, disadvantages, and dissection. Note: References are provided within the table where applicable.

Flap	Advantages	Disadvantages	Dissection
Dorsal ulnar flap (Becker and Gilbert flap)	Large skin paddle (10-20 × 5-10 cm). Does not compromise the ulnar artery. Can be used for both dorsal and volar defects, even reaching the metacarpophalangeal joints. The position of the dorsal ulnar artery is constant, making it safe. Good venous drainage. Allows for immediate hand mobilization [6,10-13].	Short pedicle, 2-3 cm. Risk of ulnar nerve injury. Poorly innervated and therefore not very sensitive [6,10-12].	Quick and easy [11,18].
Reverse radial forearm flap	Large skin paddle (10 × 20 cm). Can cover dorsal and volar defects. An even longer pedicle can be obtained by making an incision in the intermetacarpal space to reach metacarpophalangeal defects [6,13].	The radial artery and its branches are sacrificed. The anterolateral aesthetic defect is usually worse compared with the dorsal ulnar flap [6,11,12].	Quick and easy [18].
Reverse posterior interosseous flap	6-7 cm skin paddle. No sacrifice of major arteries. Primarily used for dorsal defects, although it can also be used in the wrist and hand. Can be used when other flaps are contraindicated [6,13].	Risk of posterior interosseous nerve injury. Viability may be questionable, with risks of necrosis and frequent venous congestion [6,12].	Challenging [11,12].
Lateral antebrachial neurocutaneous flap	Moderate skin paddle (10 × 5 cm). Long pedicle. No sacrifice of major arteries. Allows coverage of volar, dorsal, metacarpophalangeal joint, and thumb defects. May preserve sensation. Low risk of congestion [13,15].	Mild sensory deficit [15].	Easy [15].
Groin flap (McGregor flap)	Very large skin paddle (30 cm). Provides extensive coverage for medium and large defects. A reliable option when follow-up may be uncertain. Can cover any hand and wrist defect. Donor scar is aesthetically acceptable [6,17].	Requires prolonged hand immobilization for 3 weeks. Requires two surgeries. Due to its position, it tends to develop edema and infection. Up to half of patients may develop groin pain [6,16,17].	Easy [17].
Thoracoepigastric flap	Provides broad coverage for large defects. A reliable option when follow-up may be uncertain. Avoids hand edema, as it is positioned higher than the inguinal flap. Can cover any defect of the forearm, wrist, and hand. Can be combined with tissue expanders [16].	Requires prolonged hand immobilization for 3 weeks. Requires two surgeries [16].	Easy, but may require large incisions [16].

**Table 2:** Pedicled flaps for coverage of defects in the distal forearm and wrist: vascular supply, pivot point, donor-site morbidity, varieties, and contraindications. Note: References are provided within the table where applicable.

Flap	Vascular supply	Pivot point	Donor-site morbidity	Varieties	Contraindications
Dorsal ulnar flap (Becker and Gilbert flap)	Dorsal ulnar artery, a branch of the ulnar artery [11,18].	Approximately 3-5 cm from the pisiform bone [10,13,18].	Can be closed by primary intention for 3-4 cm flaps or may require grafting [10,11].	Fasciocutaneous and aponeurotic [10,11].	Atherosclerosis, occlusion, or injury of the proximal ulnar artery. Previous surgeries on the flap pedicle [10,12].
Reverse radial forearm flap	Radial artery [6].	Radial styloid process [6].	Can be closed by primary intention or may require grafting [6].	Fasciocutaneous, aponeurotic, and osteoseptocutaneous [6].	Abnormal Allen test [6].
Reverse posterior interosseous flap	Anastomosis between the posterior interosseous artery and the anterior interosseous artery, a branch of the ulnar artery [6,13].	Approximately 2-3 cm from the distal radioulnar joint, near the ulnar styloid process [6,13].	May be closed by primary intention or may require grafting.	Fasciocutaneous [6,13].	Should be avoided when there is significant injury to the wrist or forearm because of the risk of thrombosis of the posterior interosseous artery [6]. Previous surgeries on the flap pedicle.
Lateral antebrachial neurocutaneous flap	Intrinsic and extrinsic arterial plexus surrounding the lateral antebrachial cutaneous nerve [13,15].	Approximately 5 cm from the radial styloid process [13,15].	May be closed by primary intention or may require grafting [15].	Fasciocutaneous [13,15].	Previous surgeries on the flap pedicle.
Groin flap (McGregor flap)	Superficial circumflex iliac artery [6,17].	2 cm from the inguinal ligament [17].	Despite its size, it can be closed by primary intention [6,17].	Fasciocutaneous [6,17].	No particular contraindications.
Thoracoepigastric flap	Thoracoepigastric artery [16].	On one side of the torso, along a line drawn between the scapula and the umbilicus [16].	Despite its size, it can be closed by primary intention [16].	Fasciocutaneous [16].	No particular contraindications.

Although the original article by Becker and Gilbert presents eight cases in which the use of the dorsal ulnar flap was described for the first time, none involved reconstruction following CS, fasciotomy, crush injuries, or burns [11].

To put things into perspective, in a study in which this technique was applied to 10 patients over a 10-year period, all patients who underwent the procedure were successfully treated; the only drawback was that in one-fifth of the patients, an aesthetic result could not be achieved because grafts were required from the donor site [10].

A case series conducted by Delgado-Ruiz et al. involved patients who suffered electrical burns; three of these patients developed CS and required subsequent fasciotomy. They subsequently required a pedicled dorsal ulnar flap. The flap surgeries were performed 27 to 38 days later, achieving coverage of the wrist in all cases and allowing for subsequent procedures without complications [18].

Pizarro-Amigo et al. presented five case studies of this flap. In their series, four patients had a satisfactory post-flap outcome; however, in one case, the flap was lost due to infection, and another patient presented with partial epidermolysis without requiring flap removal. Nevertheless, all patients subsequently returned to their activities with satisfactory results [12].

We present a case of CS following entrapment of the upper limb in a dryer, involving both thermal and crush mechanisms, which synergistically produced a deleterious effect and caused greater damage. Furthermore, the severity of the burn may have been initially underestimated, since the surgical findings suggested a third-degree burn, or even a fourth-degree burn. As a few days passed, we observed necrotic patches in the muscle and tendons, as well as complete involvement of the phalanges. In any case, it is very difficult to determine how much tissue damage was caused by a specific mechanism, since the mechanisms occurred in combination. In addition, our patient also underwent delayed fasciotomy (>12 hours), which required a dorsal ulnar flap as a reconstructive procedure to achieve skin coverage of the wrist and hand, as well as amputation of the distal phalanges. We needed a safe, accessible procedure for our patient that would allow early rehabilitation. The dorsal ulnar flap proved to be an ideal procedure for our patient, and morbidity was minimal because both arterial axes of the hand were preserved.

We recognize that closing fasciotomies is complex; tertiary intention closure, negative pressure therapy, and staged closure are valid alternatives. In our case, we opted for the dorsal ulnar flap, as this technique provides good coverage of the wrist and hand without compromising distal blood flow; furthermore, its dissection is simple and quick, while also facilitating mobility and early rehabilitation. While the use of fasciotomies to treat CS is well established, the same cannot be said for the methods used to close them; in fact, studies on the use of the dorsal ulnar flap in these cases remain very scarce even today. We hope that our clinical case will contribute to the body of evidence from studies on the treatment of CS and the closure of fasciotomies following such treatment and, why not, provide a valid framework to guide decision-making for those who find themselves at a crossroads, just as we did at the time.

## Conclusions

CS requires a high index of suspicion to ensure timely diagnosis; this diagnosis should initially be clinical, and in uncertain cases, invasive methods with continuous monitoring should be used to avoid delays in performing fasciotomy, thereby preventing complications and, above all, limb loss.

After fasciotomy, the subsequent procedures required to achieve wound closure are equally important. In this scenario, the dorsal ulnar flap has proven to be an effective and easy-to-perform procedure that preserves the main arterial axes of the hand and can be performed in a single intervention to cover wounds with tendon exposure in the wrist and hand, unlike other pedicled flaps that do not have these advantages.
